# Point-of-Care Ultrasonography Helped to Rapidly Detect Pneumomediastinum in a Vomiting Female

**DOI:** 10.3390/medicina59020394

**Published:** 2023-02-17

**Authors:** Yun-Hao Chen, Po-Chen Lin, Yu-Long Chen, Giou-Teng Yiang, Meng-Yu Wu

**Affiliations:** 1 Department of Emergency Medicine, Taipei Tzu Chi Hospital, Buddhist Tzu Chi Medical Foundation, New Taipei 231, Taiwan; 2 Department of Emergency Medicine, School of Medicine, Tzu Chi University, Hualien 970, Taiwan

**Keywords:** pneumomediastinum, Boerhaave syndrome, vomiting, subcutaneous emphysema

## Abstract

Vomiting-induced pneumomediastinum is a rare presentation and can be a result of alveolar rupture (Mackler effect) or Boerhaave syndrome. Patients diagnosed with Boerhaave syndrome may present with the classic Mackler triad of vomiting, chest pain, and subcutaneous emphysema. However, there exists a large overlap of symptoms accompanying Boerhaave syndrome and the Macklin effect, including retrosternal chest pain, neck discomfort, cough, sore throat, dysphagia, dysphonia, and dyspnea. Boerhaave syndrome is a dangerous condition. Delayed diagnosis of Boerhaave syndrome may worsen sepsis and cause mortality. Therefore, early diagnosis and timely management are important to prevent further complications. Here, we present a case of vomiting-induced pneumomediastinum, which supports the use of bedside ultrasonography to aid in the diagnosis and rapid differentiation of etiology of pneumomediastinum.

## 1. Introduction

Pneumomediastinum is a condition in which air accumulates in the mediastinum. Pneumomediastinum may be caused by air escaping from the lungs, airways or hollow organs into the chest cavity due to trauma injury or other conditions. The overall incidence is reported as 1/25,000 cases in ages 5–34 years and 76% of cases are males [1,2]. Pneumomediastinum frequently occurs as a result of blunt trauma or esophageal perforation, but it is difficult to differentiate the etiology of Boerhaave syndrome and the Macklin effect due to similar symptoms. The Macklin effect was described in 1944 by Macklin et al. [3] to outline the mechanism of respiratory pneumomediastinum. Leaking air from a rupture site of alveoli or bronchioles centripetally dissects through the pulmonary interstitium along the bronchovascular sheaths into the mediastinum. The common triggers are asthma, vomiting and the Valsalva maneuver.

Boerhaave syndrome is a rare life-threating condition with a higher mortality rate [4,5]. Rupture of the esophagus from severe straining or vomiting can progress to emphysema, mediastinitis and septic shock, especially in delayed diagnosis [5,6]. In an analysis of 24 patients with spontaneous pneumomediastinum, the most common symptoms were chest pain, neck discomfort, and cough [7]. In another report of 17 patients with spontaneous pneumomediastinum, 82% of patients presented with chest pain and dyspnea, but only 18% of patients presented with neck discomfort [8]. The lower third of the esophagus and the left lateral position is the most common site in 90% of cases. The mortality rate is 8–60%, with higher risk of morbidity [2,3,4]. Early diagnosis is vital to prevent mortality and morbidity. However, in the early stages of Boerhaave syndrome, a patient may present with nonspecific symptoms due to a smaller amount of leaking air, contributing to a delay in diagnosis. Because of the nonspecific presentation that can mirror other disorders, the diagnosis of Boerhaave syndrome is challenging and can often be overlooked [9]. In this article, we provide a case who presented with vomiting-induced subcutaneous emphysema and pneumomediastinum which was detected early by point-of-care ultrasonography to highlight Boerhaave syndrome and the Macklin effect.

## 2. Case Presentation

**Figure 1:**
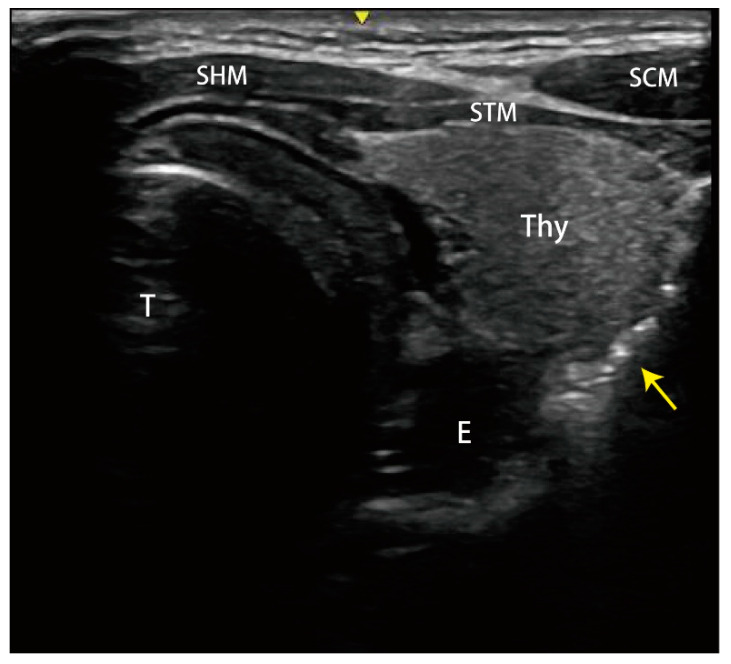
Point-of-care ultrasound found the free air (yellow arrow) in the soft tissue of the neck with ring-down artifacts along the esophageal tract. STM: sternothyroid muscle; SHM: sternohyoid muscle; SCM: sternocleidomastoid muscle; E: esophagus; T: trachea; Thy: thyroid gland.

**Figure 2:**
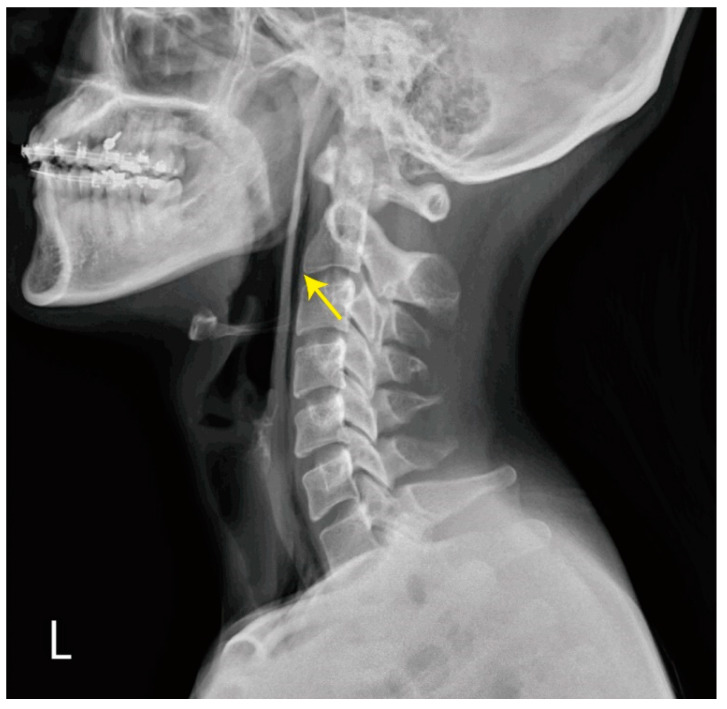
Chest X-ray showed subcutaneous emphysema over the neck and gas outlining from neck extending into the mediastinum (yellow arrow).

**Figure 3:**
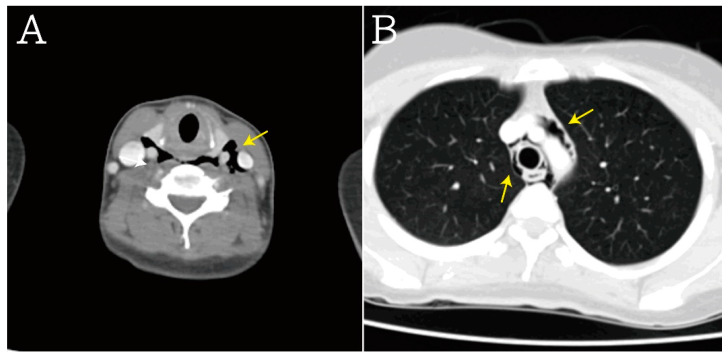
In computed tomography scans, these results were also noted, including (**A**) subcutaneous emphysema (yellow arrow), (**B**) pneumomediastinum, periaortic gas, and pericardiac gas (yellow arrow).

**Figure 4:**
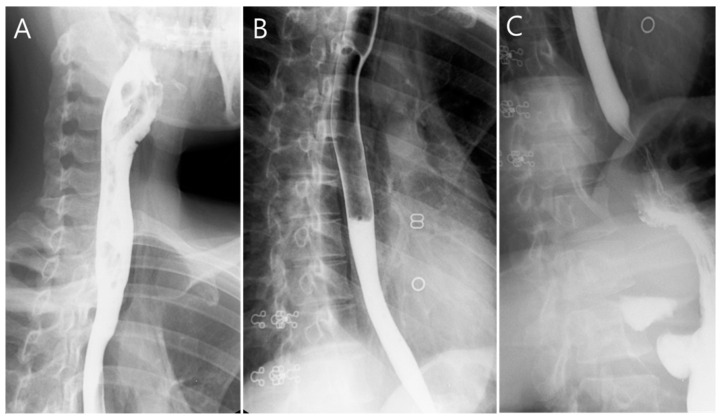
Upper gastrointestinal series revealed no obstruction and leakage at (**A**) upper, (**B**) middle, and (**C**) lower esophagus.

**Figure 5:**
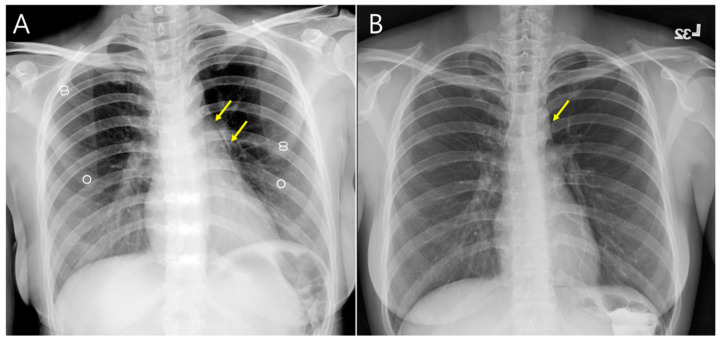
Sequential chest X-ray (**A**) on admission day and (**B**) discharge day showed improvement of pneumomediastinum (yellow arrow). There was no pleural effusion and pericardial effusion.

A 27-year-old woman without trauma or previous surgical history presented to the emergency department with sudden-onset neck pain and mild dysphagia after postprandial vomiting. She was restless and clutching her hands to her throat. There was no traumatic history or lung disease. On physical examination, she was alert, slightly tachycardiac at 104 beats per minute, and her other vital signs were as follows: blood pressure 118/53 mmHg, respirations 17 breaths per minute and pulse oximetry 99% on room air. Palpable crepitus was found in the patient’s neck region without tenderness. Laboratory tests revealed leukocytosis (WBC count: 19.61 × 10^3^/µL, normal range: 3.5–11.0 × 10^3^/µL), but C-reactive protein level, troponin-I, and the other results of laboratory tests were within normal limits. Point-of-care ultrasound (POCUS) of the neck region was performed. Figure 1 and Video S1 visualize the free air in the soft tissue of the neck with ring-down artifacts along the esophageal tract. A neck radiograph was used to establish the diagnosis, as seen in Figure 2, of subcutaneous emphysema over the neck and pneumomediastinum with continuous air dissecting the posterior pharyngal wall. The diagnosis was confirmed by contrast-enhanced computed tomography. Figure 3 shows subcutaneous emphysema pneumomediastinum. We closely monitored the breathing pattern to prevent pneumothorax, tension pneumomediastinum, and tension pneumopericardium due to increased pressure in the mediastinal cavity, which may potentially cause serious complications, such as airway obstruction. A broad-spectrum antibiotic agent was administrated to prevent infection. An upper GI series was performed to rule out Boerhaave syndrome as shown in Figure 4. There was no leakage or obstruction at the upper, middle, and lower esophagus. A sequential chest X-ray, as shown in Figure 5, revealed improvement of pneumomediastinum and decreased air leakage. The patient was discharged without any complications.

## 3. Discussion

POCUS is a method of rapid evaluation of pneumomediastinum with minimal radiation exposure and helps lead to early diagnosis of certain aspects of the syndrome, such as mediastinal or free peritoneal air or subcutaneous emphysema. In 1983, the sonographic evaluation of pneumomediastinum was reported and mentioned the “air gap sign”, which is described as accumulating air obscuring normal cardiac structures [10,11]. Sonographic findings in pneumomediastinum include (1) visualized free air in the soft tissue of the neck demonstrating hyperechoic foci with ring-down artifacts; (2) the air gap sign demonstrating air within the mediastinum or pericardium that obscures normal cardiac structures. Air artifact or A-lines with loss of the parasternal and apical views may be seen when performing transthoracic echocardiography, but the subxiphoid view remains clear in pneumomediastinum, which may help differentiate from pneumopericardium [12,13]. POCUS can be used to carry out a quick initial workup for those who are not suitable for computed tomography, such as hemodynamically unstable patients or pregnant women.

Lung structure damage due to fibrosis, infection, or inflammation may increase the risk of pneumomediastinum, such as COVID-19 infection. Patients with COVID-19 pneumonia have higher risk of pneumomediastinum under mechanical ventilation. Rodrigo Gobbo Garcia et al. [14] reported that tension pneumomediastinum was found in a 60-year-old man with COVID-19 under mechanically ventilation for 32 days. The tension pneumomediastinum with compressed cardiac chambers simulating acute cardiac tamponade was diagnosed by a CT scan in a negative pressure interventional suite. However, this facility is not widely available in all hospitals. POCUS is a useful bedside diagnostic tool to minimize the risk of cross-infection related to COVID-19 patient transport.

The pediatric population is considerably more sensitive to radiation than adults. POCUS is more feasible for the diagnosis of neonatal pneumomediastinum. Children with neonatal pneumomediastinum may present with signs of respiratory distress and muffling of cardiac sounds. These children need to be closely observed to determine the severity of pneumomediastinum. In Erik Küng et al.’s report [15], the classic configuration of pneumomediastinum in ultrasound is described as the “angel-wing” or “spinnaker sail” thymus due to air loculated within the anterior mediastinum. Additionally, a stairway-like arrangement of horizontal hyperechogenic reflections may be found in lung ultrasound parasternal view when the air is trapped below the thymus.

Chest and cervical radiography can also be supportive of the diagnosis. Chest radiograph is usually used to establish the diagnosis of pneumomediastinum and subcutaneous emphysema. A typical pattern of a chest X-ray may show subcutaneous emphysema and pneumomediastinum with a continuous diaphragm sign. Trapped air may outline the mediastinum, aorta, and heart extending into the neck [16].

With cervical esophageal perforations, neck radiography may reveal free air dissecting along the soft tissues of the prevertebral space. Other findings suggestive of an esophageal perforation include pleural effusions (usually left-sided), pneumopericardium, hydropneumothorax, or subdiaphragmatic air [17]. The diagnosis of pneumomediastinum is most commonly based on chest radiographs and established by contrast esophagram or computed tomography scans, which may reveal extravasated contrast or free air surrounding the esophagus or in the mediastinum. However, there are cases of false-negative results the severity of the SPM is underestimated in 10–30% of cases [18,19,20].

A CT scan is considered to be the preferred imaging for pneumomediastinum, especially in esophageal emergencies [21,22]. CT scans provide detailed information for the diagnosis of pneumomediastinum and to differentiation from Boerhaave syndrome and the Macklin effect. In Boerhaave syndrome, esophageal wall thickening and defect, and periesophageal fluid and/or air, may be found. The Macklin effect appears on CT as linear collections of air contiguous to the bronchovascular sheaths. The air dissects into the pulmonary hila and from there enters the mediastinum. Pneumomediastinum is generally considered a typically benign presentation with good prognosis. Treatment for pneumomediastinum is generally conservative, but the extent of treatment is dependent on severity. A large amount of air entrapped in the mediastinum, known as malignant pneumomediastinum, may cause blockage of the trachea and vessels. Emergent decompression with thoracotomy should be considered in these patients.

## 4. Conclusions

Our report shows the sonographic features of pneumomediastinum in a 27-year-old woman who had been vomiting, which supports the use of POCUS to help rapid diagnosis of this less common cause of neck or chest pain. When evaluating uncommon neck or chest pain, pneumomediastinum should be suspected when POCUS demonstrates free air in the periesophageal soft tissue. We believe that ultrasound will be used more commonly and easily to rapidly evaluate pneumomediastinum in clinical practice.

## Data Availability

All data and material are presented in the manuscript.

## Supplementary Materials

The following supporting information can be downloaded at: https://www.mdpi.com/article/10.3390/medicina59020394/s1, Video S1: Video for POCUS.
